# Invasion of Calcium Hydroxide Preparations Leading to Severe Chemical Nerve Injury Treated Through Nerve Repair Using Artificial Nerve Conduit: A Case Report

**DOI:** 10.1155/crid/6610331

**Published:** 2025-12-12

**Authors:** Akihiro Nishiyama, Andreas Neff, Takahiro Nakada, Takaharu Ariizumi, Akira Iwasaki, Keisuke Sugahara, Akira Katakura

**Affiliations:** ^1^ Department of Oral Pathobiological Science and Surgery, Tokyo Dental College, Tokyo, Japan, tdc.ac.jp; ^2^ Department of Oral Craniomaxillofacial Surgery, University Hospital Marburg, Marburg, Germany, ukgm.de; ^3^ Department of Oral and Maxillofacial Surgery, Tokyo Dental College, Tokyo, Japan, tdc.ac.jp

**Keywords:** calcium hydroxide, endodontic treatment, functional sensory recovery, inferior alveolar nerve injury, nerve repair

## Abstract

Calcium hydroxide is an effective agent in endodontic treatment worldwide. Some cases reported that treating a mandibular molar can lead to severe incidents of overfilling from the root canal, which leads to nerve disturbance. However, it remains unclear how nerve disturbance affects the degree of the recovery of sensation, whether through treatment or observation. We present a nerve repair of the inferior alveolar nerve injury caused by endodontic treatment, wherein perception did not improve after several months of observation. Postoperative pathological findings revealed that the injured nerve bundle contained calcium hydroxide and colloidal foreign bodies as a residue of barium sulfate. Therefore, sensory malfunction in our case was attributed to two factors: chemical damage due to the strong alkalinity and radiopacity of barium sulfate residue in the ingredients of calcium hydroxide and physical damage from the medication. This incident highlights the necessity for dentists to carefully handle the injection type of chemical agents for endodontic treatment or consider indirect application in the root canal. Furthermore, in the absence of recovery of sensory function, nerve repair through an artificial nerve conduit using an appropriate nerve agent should be considered to relieve pressure on the nerve. In this case of chemical injury to the inferior alveolar nerve treated with nerve repair, the nerve recovery was assessed as Grade S3 on the British Medical Research Council scale 6 months postoperatively; additionally, the nerve regained functional sensory recovery from this grade.

## 1. Introduction

Root canal treatment, which is a common treatment procedure in endodontics, requires materials to clean and fill the root canal. Calcium hydroxide is widely used in endodontics due to its effective bacteria‐reducing properties and its characteristics as a safe, solid alkali material [1]. Therefore, it is more convenient for dentists to use calcium hydroxide as an injection type. In Japan, there are two main types: Calcipex II (Morita, Osaka, Japan), which contains calcium hydroxide (24%) and barium sulfate (24%), and Vitapex (DiaDent Group International Inc., Burnaby, BC, Canada), which contains calcium hydrate (30%), iodoform (40.4%), and silicon oil (22.4%). These materials can lead to issues such as overfilling from the apex of the root canal since the piston of the syringe is pushed with high pressure, which potentially damages the tissues around the teeth. Recent articles have also focused on endodontic treatment for mandibular molars causing inferior alveolar neuropathy. The peripheral sensory nerve injured by chemical agents causes a disturbance of perception.

Additionally, some articles reported that the inferior alveolar nerve (IAN) regained its perception from neuropathy after several weeks to several years. Hence, in most recovery cases, perception was similar to that of the first endodontic treatment. Some cases reported conservative treatments to remove the agent by irrigating the root canal using normal saline or observing until foreign bodies were absorbed and perception had recovered. However, all cases necessitated clarification regarding the evaluation of perception and the details of the procedure for recovering perception. Moreover, it remains unclear whether nerve injury occurred due to chemical agents. In this case, we report a nerve repair of IAN injury due to endodontic treatment. The perception did not improve after observing this condition for several months. Additionally, we considered this nerve injury a pathological and clinical disturbance.

## 2. Case Presentation

A 45‐year‐old Japanese woman underwent root canal treatment for the left mandibular second molar owing to chronic apical periodontitis at the dental clinic. After the initial treatment, sensory disturbance in the left lower alveolar nerve area occurred. At 16 days after the onset of symptoms, the patient was referred to Tokyo Dental College Suidobashi Hospital owing to the persistence of disturbance. The patient′s medical history was unremarkable. As a nerve injury of the IAN related to local anesthesia or materials used in the endodontic treatment was considered, several inspections were performed to diagnose the patient. Panoramic x‐ray and CT images revealed overflowing of calcium hydroxide using an endodontic agent from the apical foramen of the distal root of the lower left second molar in the mandibular canal (Figure 1). During the evaluation of the nerve perception, the Semmes–Weinstein (SW) monofilament indicated a maximum value of 100 g, making it difficult to determine the degree of perception. The static two‐point discrimination threshold (s2PD), temperature, and pain sensation were also absent (Table 1). Finally, pushing with high pressure using a Calcipex II syringe type during the treatment was revealed, according to the previous doctor. Therefore, it was concluded that the left IAN was damaged by calcium hydroxide, leading to nerve injury caused by a chemical agent. Hence, the previous doctor was requested to irrigate the root canal and remove the agent prior to deciding the treatment plan. While diagnosing the degree of neurosensory impairment, conservative treatment using vitamin B12 and performing stellate ganglion block in our hospital failed to improve the condition of the left side of the chin. Additionally, the value of the SW test monofilament worsened to 180 g, and the degree of neurosensory impairment was diagnosed as comparable to partial neurotmesis according to the Seddon classification. Although surgery was the primary treatment option, the patient was consulted on selecting between continuing conservative treatment using vitamin B12, and so on, or surgical treatment. Finally, the patient decided to undergo surgical treatment.

Figure 1Panoramic x‐ray and computed tomography (CT) images at the first visit. (a) The white line indicates the mandibular canal at the second and third molars. (b) CT revealed foreign bodies to be agents of endodontic treatment.

**Figure:**
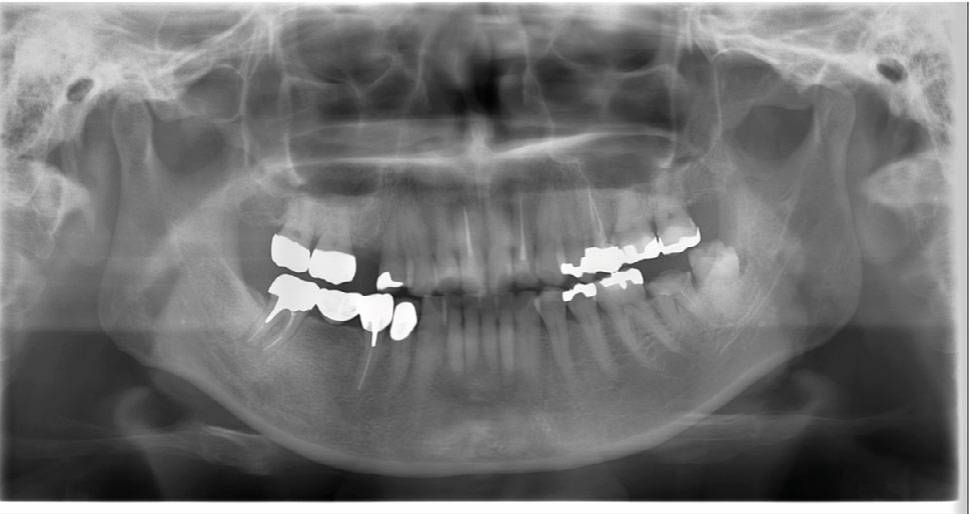
(a)

**Figure:**
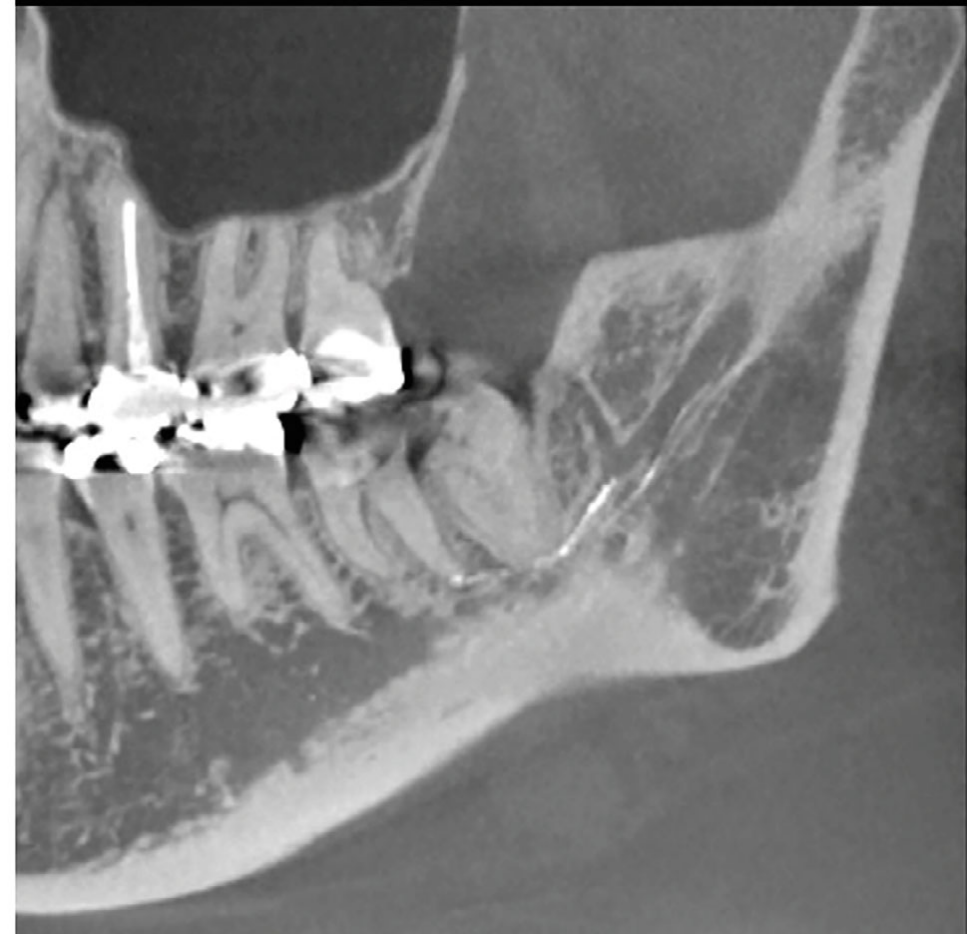
(b)

**Table 1:** The results of sensory tests preoperatively and postoperatively.

		**Healthy side**	**Affected side**					
	**Duration**		**POM-4**	**POD-1**	**POM1**	**POM3**	**POM6**	**POM11**
SW test (g/mm^2^)	Lower lip	0.008	100	180	10	2	0.16	0.16
	Chin	0.008	15	15	4	1	0.4	0.16
	Corner of mouth	0.008	0.008	0.008	0.008	0.008	0.008	0.008

s2PD (mm)		5	−	−	20	15	15	15

Thermal sensation	Warm	+	−	−	−	−	−	−
	Cold	+	−	−	−	−	−	+

Pain sensation (g)		2	−	−	−	8	8	6

Sensory disturbance	Anesthesia		○	○				
	Dysesthesia		○	○				
	Paresthesia		○	○	○	○	○	○
	Hypoesthesia		○	○	○	○	○	○
	Hyperalgesia							
	Neuralgia							

*Note:* ○, there is the symptom; +, positive; −, negative.

Abbreviations: POD, postoperative day; POM, postoperative month; s2PD, static two‐point discrimination threshold; SW test, Semmes–Weinstein test.

The surgical plan encompasses removing foreign bodies due to the overflow of Calcipex II from the IAN, along with the extraction of the left lower second and third molars under general anesthesia within 4 months after the nerve injury. It was difficult to preserve the left lower second molar since the crown was nearly collapsed and the tooth was deeply eroded within the subgingival area due to dental caries.

The mandible around the lesion was exposed, and the left lower second molar and third molar were extracted. Afterwards, a previously fabricated cutting device to determine the lesion′s extent based on imaging findings was utilized, and cortical bone removal was performed using an ultrasonic cutting instrument to reveal the IAN. The adhesive‐hardened medication was confirmed to be connected to the nerve along the course of the IAN vascular bundle (Figure 2). The medication was attempted to be removed from the nerve via normal saline irrigation; however, the medication could not be removed, indicating firm adhesion to the nerve bundle. Therefore, 29 mm of the affected nerve bundle was excised, and then an artificial nerve conduit tube, which consisted of polyglycolic acid (PGA) and collagen sponge (Nerbridge; Toyobo, Osaka, Japan), was transplanted into the defect and sutured with three threads made of propylene (Necosuture; Alfresa, Osaka, Japan) on each side. Subsequently, the cortical bone was repositioned and fixed with a 1.0 mm titanium plate and seven screws (KLS Martin SE & Co. KG, Tuttlingen, Germany) (Figure 3). Finally, the left lower second molar was extracted, and the procedure was concluded with closure. The overflowed foreign bodies and nerve bundle were firmly adhered together, making simple irrigation ineffective. Therefore, the specimens were sent for pathological examination. Pathological findings revealed irreversible degeneration of structures resembling nerve fibers due to infiltration by calcium hydroxide preparations. Additionally, colloidal foreign bodies were identified, which may potentially be residue of barium sulfate from Calcipex II (Figure 4). Postoperatively, stellate ganglion block was done once a week for a total of 20 times (up to 2 months after the operation), and vitamin B12 preparation (1500 *μ*g/day) was given for 12 months after the operation for the IAN. Additionally, steroid administration was tapered postoperatively. No side effects of the medicine or severe postoperative complications were observed. At 11 months postoperatively, despite the persistence of some paresthesia and hypoesthesia at the innervation of IAN, sensory tests indicated an improvement compared to the preoperative condition (Table 1). According to the British Medical Research Council scale for grading sensation, the postoperative scoring scale was graded as S3 (Table **2**), and it acquired functional sensory recovery (FSR) (S3, S3+, and S4) after 6 months postoperatively [2].

Figure 2Approach to the inferior alveolar nerve. (a) Setting the cutting device. (b) Cutting the cortical bone. (c) Removal of the cortical bone and view of the inferior alveolar nerve with calcium hydroxide.

**Figure:**
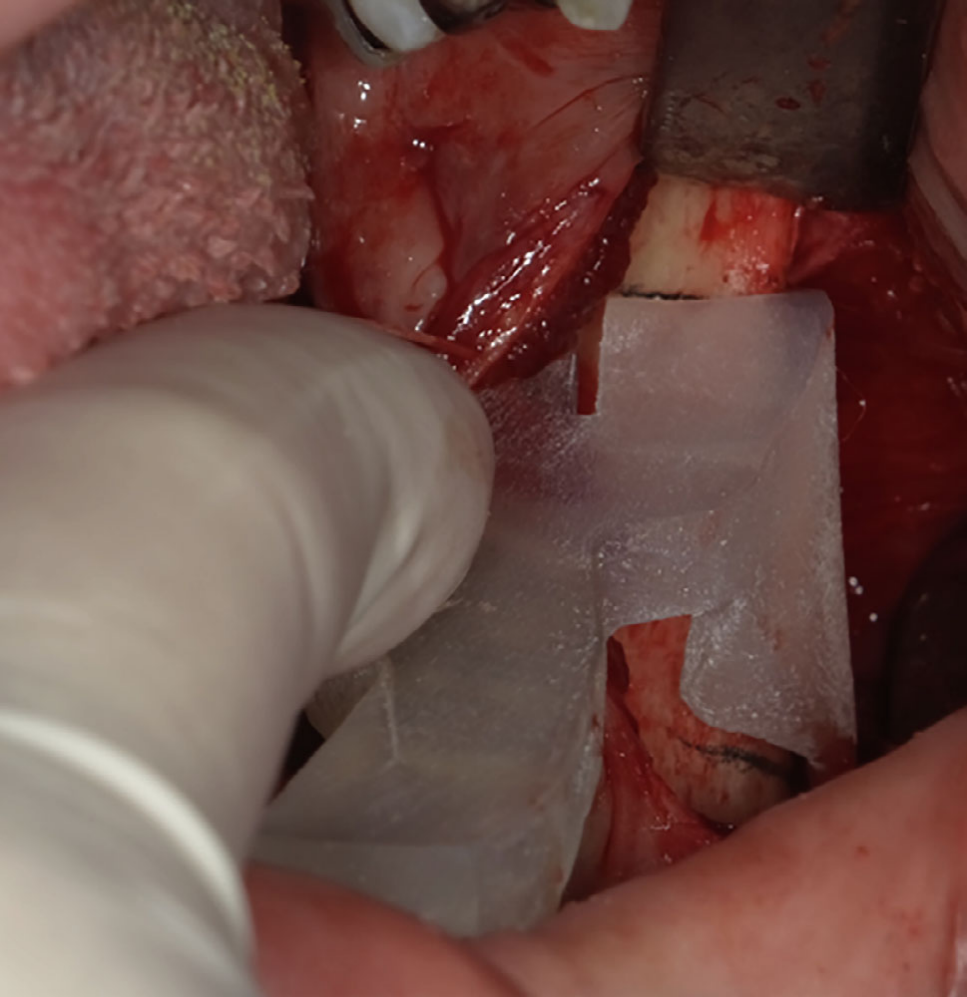
(a)

**Figure:**
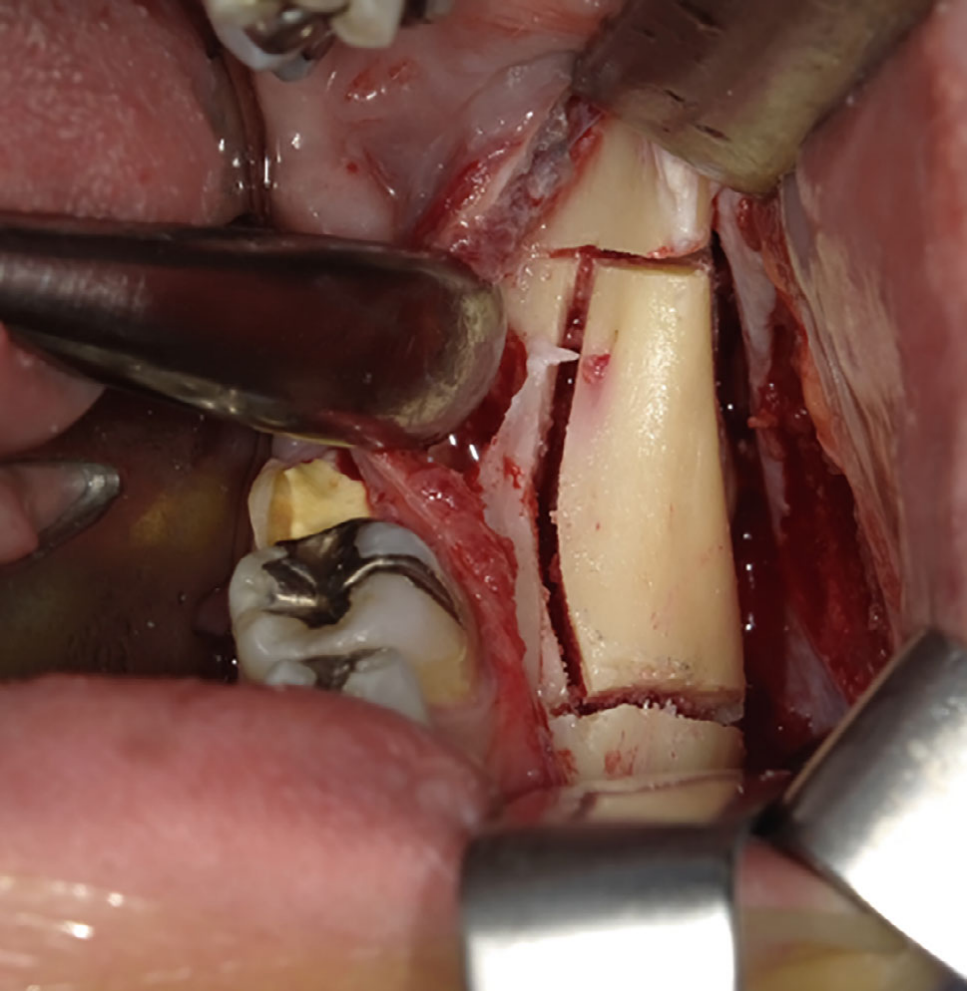
(b)

**Figure:**
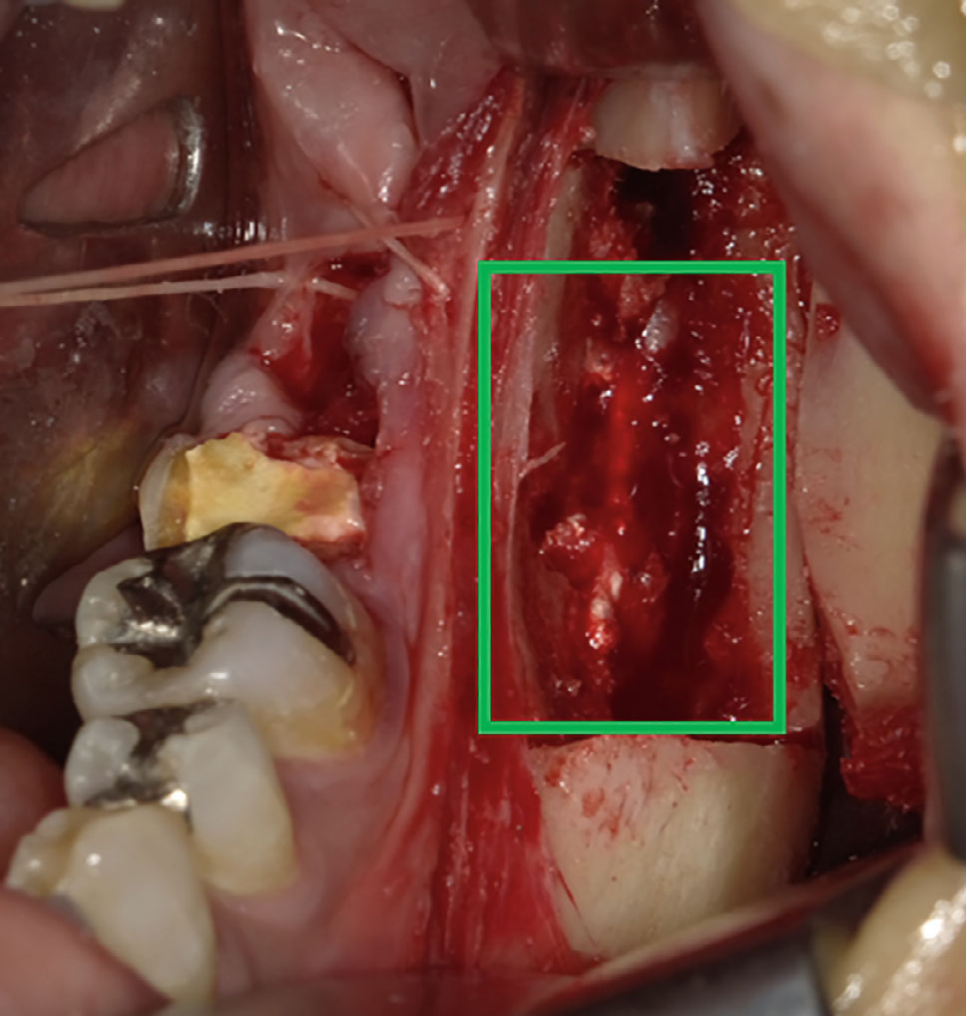
(c)

Figure 3Nerve repair. (a) Grafting with Nerbridge. (b) Repositioning and fixation of the cortical bone using a metal plate. (c) Closure of the wound.

**Figure:**
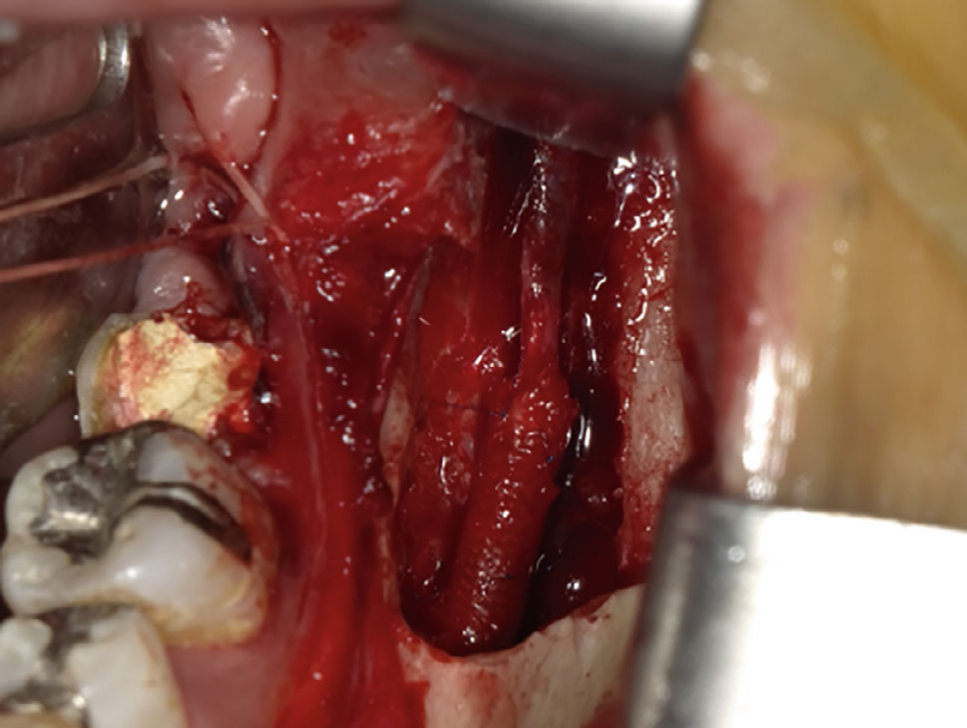
(a)

**Figure:**
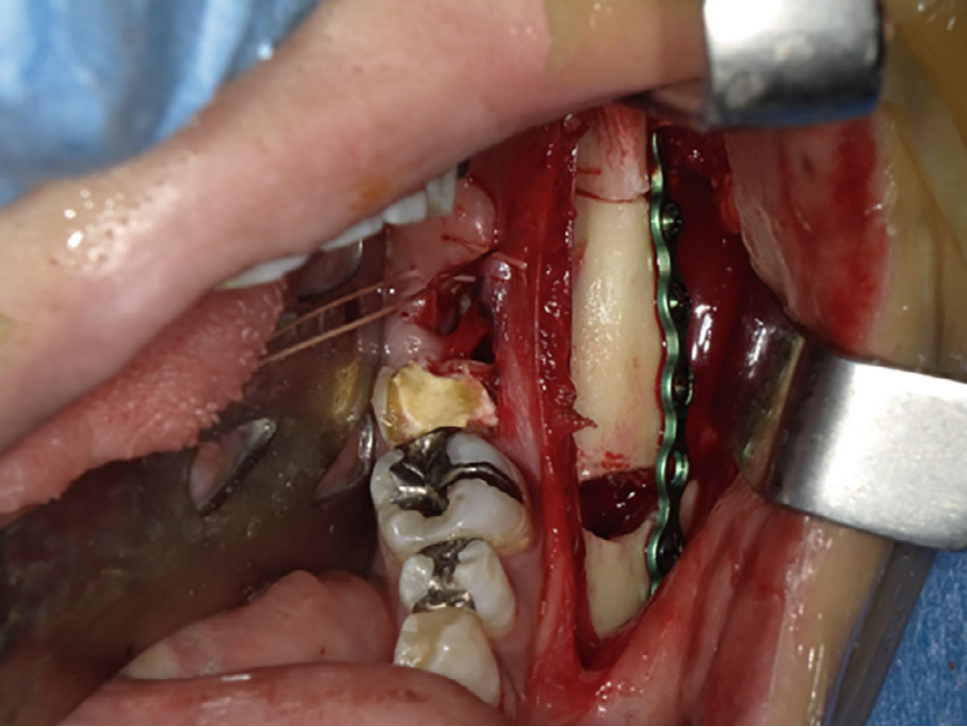
(b)

**Figure:**
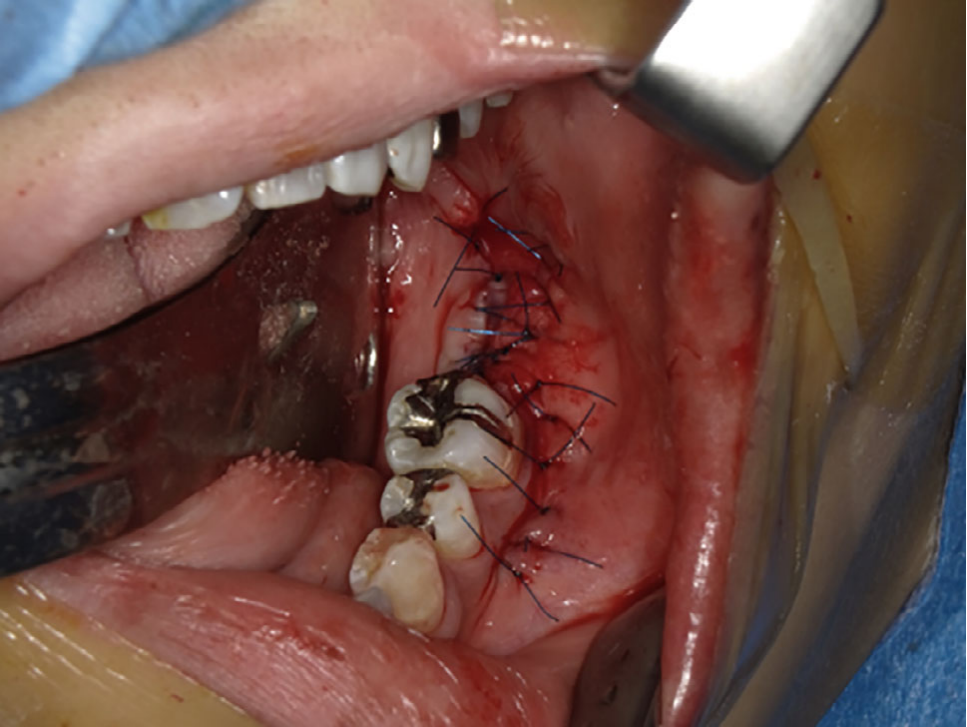
(c)

Figure 4Specimen for pathology. (a) The inferior alveolar nerve specimen surrounded by calcium hydroxide preparations and fibroadipose tissue. (b) Hematoxylin and eosin stain, original magnification: 40×. (c) The nerve bundles around the drug were chemically degenerated, with the yellow arrow indicating residue of barium sulfate. H&E stain, original magnification: 100×.

**Figure:**
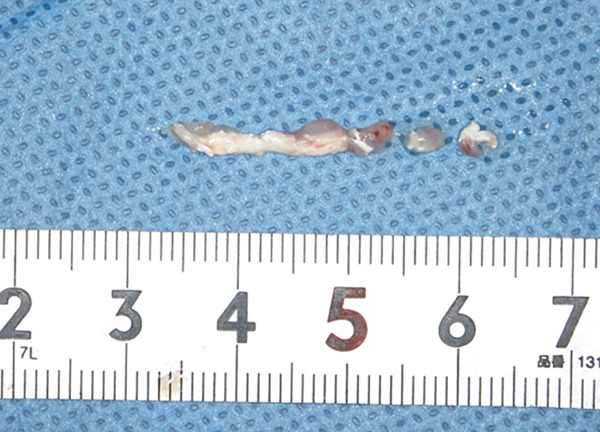
(a)

**Figure:**
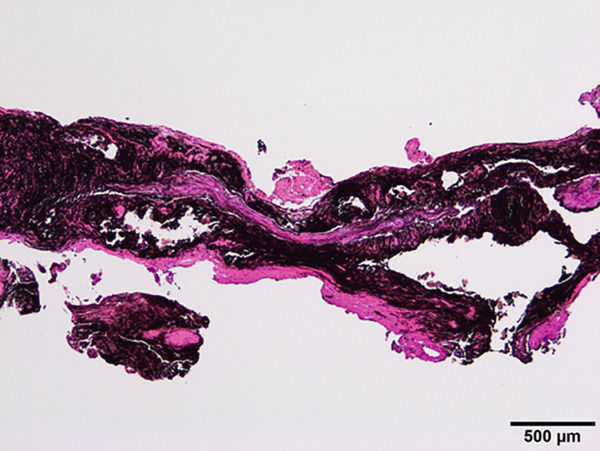
(b)

**Figure:**
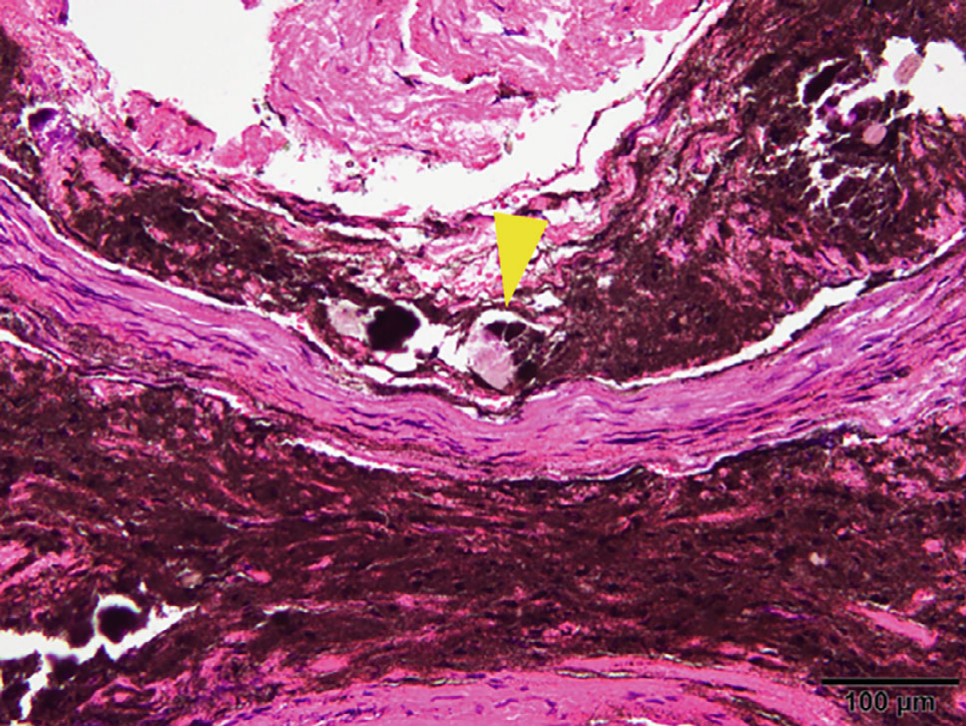
(c)

**Table 2:** Medical Research Council scale.

**Grade**	**Description**
S0	No sensation
S1	Deep cutaneous pain in autonomous zone
S2	Superficial pain and touch
S2+	Superficial pain and touch add hyperesthesia
S3+	Superficial pain and touch without hyperesthesia; s2PD > 15 mm
S3	Same as S3 with good stimulus localization and s2PD of 7–15 mm
S4	Same as S3 with good stimulus localization and s2PD of 2–6 mm

Abbreviation: s2PD, static two‐point discrimination threshold.

## 3. Discussion

Kasapoğlu and Doğancalı reported cases of IAN damage due to Vitapex. Six months after neurosensory injury, Vitapex was completely absorbed on x‐ray, and 3 years later, a complete disappearance of sensory numbness was reported without surgery [3]. The case suggests that not only the degree of disturbance or nerve injury such as axonotmesis in the Seddon classification but also the differences in the content and absorbability of the agent may influence the pathophysiological changes of nerve damage compared to Calcipex. Particularly, barium sulfate residue as a contrast agent in Calcipex may also exacerbate peripheral nerve injury as compared with iodoform in Vitapex. Therefore, the choice of agent (Calcipex or Vitapex) may influence the degree of nerve injury [4].

Shin et al. reported paresthesia of the IAN upon performing endodontic treatment using Calcipex for nerve repair at 2 months after nerve injury. However, sensory function did not improve 20 months postoperatively. Hence, they suggested practicing caution when using the injection type of chemical agent for endodontic treatment to prevent nerve injury and recommended using alternative methods for administering the agent for a kind of lentulo [4]. Regarding the pressure on the nerve, Yatsuhashi et al. reported the effectiveness of immediate removal of root canal filling materials via cleaning with a microscope‐reduced decompression and chemical damage within the inferior alveolar canal, thereby suppressing irreversible nerve damage [5]. Pogrel reported that clinicians must monitor patients for a 72‐h period following local anesthesia, even when the anesthetic effect seems to resolve adequately with normal sensation recovery. This is necessary because nerve injury with delayed onset may occur with agents having lower neurotoxicity. Nerve decompression, debridement, thorough irrigation, and cleansing should be implemented immediately in the event that symptoms emerge at the time when the local anesthetic effect is expected to have dissipated, as this approach has the potential to produce optimal outcomes [6]. These articles highlighted the variety of sensory disturbances in endodontic therapy, including paresthesia, dysesthesia, and pain. In addition to those caused by calcium hydroxide preparations, agents such as paraformaldehyde are examples of pulp devitalizing, gutta‐percha, and root canal sealers. Among these, nerve disturbance due to calcium hydroxide preparations has been reported in four cases. Three cases revealed complete recovery from sensory disturbance, while one case showed partial recovery within the observation period [4–16].

In our case, the malfunction of the sensory was caused by two factors: chemical damage from the strong alkalinity and radiopacity of barium sulfate residue in the calcium hydroxide preparations and physical damage caused by the medication being forcefully pushed into the inferior alveolar canal, increasing pressure within the canal and compressing the IAN. These findings were confirmed via pathological examination findings. According to these findings, chemical damage to the peripheral sensory nerve caused by the medication, especially considering its expansion and sustained release characteristics, is expected to be more severe compared to physical damage, such as that caused by wisdom tooth extraction. Regarding neurosensory recovery after surgical treatment, Byun et al. reported performing nerve repair surgery for nerve damage caused by root canal filling material, and FSR through nerve repair surgery depends on the extent of damage and the immediate removal of the causative agent. In the early repair group, where surgery was performed within 60 days of injury, the average time for FSR was reported to be 198 days, while in the late repair group, where surgery was performed after 60 days postinjury, it was reported to be an average of 241.3 days [17]. Additionally, Schwenzer and M. Ehrenfeld recommended generally performing nerve repair for neurotmesis until 3 months postinjury [18]. In our case, although nerve repair was performed 116 days postinjury, FSR was achieved within 180 days postoperatively. Nerbridge was utilized for nerve repair due to the approximately 29 mm defect after removing the widely spread chemical agent. Surgical reconstruction was selected due to persistent paresthesia of the IAN for over 6 months and inability to absorb the medication. Additionally, the residue of barium sulfate found in the pathological specimen was indicated to induce continuing pressure and distract the agents for the whole of the peripheral nerve bundle, necessitating the removal of the affected nerve and surgical reconstruction. The achieved FSR in our case highlights the significance of completely removing the material from the IAN for the improvement of function. Hence, using artificial nerve conduit for nerve repair is considered to improve the regeneration of the peripheral nerve, especially in cases of wide invasion due to severe chemical injury, compared to neurolysis or neurorrhaphy.

Since 2015, an injection syringe for calcium hydroxide has been equipped with a safety stopper to prevent these incidents in Japan, along with cautionary measures against excessive pressure during administration. Despite changing this equipment for the agent, accidents such as in our case still occur; hence, dentists must carefully consider the use of injection‐type chemical agents for endodontic treatment or opt for indirect administration in the root canal. Additionally, dentists should understand the closest anatomical distance between the apex of the mandibular molar and the IAN [19]. This case highlights the importance of accurately diagnosing nerve injury caused by chemical agents such as calcium hydroxide used in endodontic treatment and the timing of nerve repair. In particular, when diagnosing, it is crucial to assess whether the initial injury was chemical or traumatic in nature because chemical damage can have a more severe impact on nerve function. If a nerve disturbance such as that in our case is encountered, nerve repair and immediate pressure reduction on the nerve should be performed after considering the result of sensory function at that time.

## 4. Conclusions

We encountered a case of chemical injury to the IAN, which was treated with nerve repair, resulting in perception recovery. If a similar nerve disturbance, such as that in our case, is encountered, corresponding nerve repair and immediate pressure reduction on the nerve should be performed after considering the result of sensory function at that time. Moreover, dentists must first carefully consider the use of injection‐type chemical agents for endodontic treatment and should understand the closest anatomical distance between the apex of the mandibular molar and the IAN.

## Ethics Statement

Ethics approval is not required for case reports.

## Consent

Written consent for publication was provided by the patient.

## Conflicts of Interest

The authors declare no conflicts of interest.

## Author Contributions

A.Ni.: writing—original draft, investigation, methodology, and conceptualization. A.Ne.: writing—review and editing. T.N.: data curation. T.A.: investigation. A.I.: data curation. K.S.: investigation. A.K.: writing—review and editing.

## Funding

No funding was received for this manuscript.

## Supporting information


**Supporting Information** Additional supporting information can be found online in the Supporting Information section. We used the CARE‐checklist‐English‐2013 as a reporting guideline.

## Data Availability

Date is available upon request from the authors.

## References

[1] Athanassiadis B. , Abbott P. V. , and Walsh L. J. , The Use of Calcium Hydroxide, Antibiotics and Biocides as Antimicrobial Medicaments in Endodontics, Australian Dental Journal. (2007) 52, no. 1 Suppl, S64–S82, 10.1111/j.1834-7819.2007.tb00527.x, 2-s2.0-34247542561, 17546863. 17546863

[2] Birch R. , Bonney G. , and Wynn Parry C. B. , Surgical Disorders of the Peripheral Nerves, 1998, Churchill Livingstone.

[3] Kasapoğlu M. B. and Doğancalı G. E. , Inferior Alveolar Nerve Injury due to the Extrusion of Calcium Hydroxide During Endodontic Treatment: A Case Report, Australian Endodontic Journal. (2022) 48, no. 2, 342–346, 10.1111/aej.12650, 35770929. 35770929

[4] Shin Y. , Roh B. D. , Kim Y. , Kim T. , and Kim H. , Accidental Injury of the Inferior Alveolar Nerve due to the Extrusion of Calcium Hydroxide in Endodontic Treatment: A Case Report, Restorative Dentistry and Endodontics. (2016) 41, no. 1, 63–67, 10.5395/rde.2016.41.1.63, 26877992. 26877992 PMC4751209

[5] Yatsuhashi T. , Nakagawa K. , Matsumoto M. , Kasahara M. , Igarashi T. , Ichinohe T. , and Kaneko Y. , Inferior Alveolar Nerve Paresthesia Relieved by Microscopic Endodontic Treatment, Bulletin of Tokyo Dental College. (2003) 44, no. 4, 209–212, 10.2209/tdcpublication.44.209, 2-s2.0-3843093103, 15103918. 15103918

[6] Pogrel M. A. , Damage to the Inferior Alveolar Nerve as the Result of Root Canal Therapy, Journal of American Dental Association. (2007) 138, no. 1, 65–69, 10.14219/jada.archive.2007.0022, 2-s2.0-33846591999. 17197403

[7] Kothari P. , Hanson N. , and Cannell H. , Bilateral Mandibular Nerve Damage Following Root Canal Therapy, British Dental Journal. (1996) 180, no. 5, 189–190, 10.1038/sj.bdj.4809013, 2-s2.0-0030576064, 8867623. 8867623

[8] Blanas N. , Kienle F. , and Sándor G. K. , Inferior Alveolar Nerve Injury Caused by Thermoplastic Gutta-Percha Overextension, Journal - Canadian Dental Association. (2004) 70, no. 6, 384–387, 15175118. 15175118

[9] Grötz K. A. , Al-Nawas B. , and de Aguiar E. G. , Treatment of Injuries to the Inferior Alveolar Nerve After Endodontic Procedures, Clinical Oral Investigations. (1998) 2, no. 2, 73–76, 10.1007/s007840050048, 2-s2.0-0042638664, 15490779. 15490779

[10] Yaltirik M. , Ozbas H. , and Erisen R. , Surgical Management of Overfilling of the Root Canal: A Case Report, Quintessence International. (2002) 33, no. 9, 670–672, 12666891. 12666891

[11] Fanibunda K. , Whitworth J. , and Steele J. , The Management of Thermomechanically Compacted Gutta Percha Extrusion in the Inferior Dental Canal, British Dental Journal. (1998) 184, no. 7, 330–332, 10.1038/sj.bdj.4809618, 2-s2.0-0032507388, 9599885. 9599885

[12] Ahlgren F. K. , Johannessen A. C. , and Hellem S. , Displaced Calcium Hydroxide Paste Causing Inferior Alveolar Nerve Paraesthesia: Report of a Case, Oral Surgery, Oral Medicine, Oral Pathology, Oral Radiology, and Endodontics. (2003) 96, no. 6, 734–737, 10.1016/j.tripleo.2003.08.018, 2-s2.0-0346365213. 14676765

[13] Knowles K. I. , Jergenson M. A. , and Howard J. H. , Paresthesia Associated With Endodontic Treatment of Mandibular Premolars, Journal of Endodontics. (2003) 29, no. 11, 768–770, 10.1097/00004770-200311000-00019, 2-s2.0-1542786389, 14651287. 14651287

[14] Scolozzi P. , Lombardi T. , and Jaques B. , Successful Inferior Alveolar Nerve Decompression for Dysesthesia Following Endodontic Treatment: Report of 4 Cases Treated by Mandibular Sagittal Osteotomy, Oral Surgery Oral Medicine Oral Pathology Oral Radiology and Endodontics. (2004) 97, no. 5, 625–631, 10.1016/j.tripleo.2004.01.002, 2-s2.0-2442545334. 15153877

[15] Vasilakis G. J. and Vasilakis C. M. , Mandibular Endodontic-Related Paresthesia, General Dentistry. (2004) 52, no. 4, 334–338, 15366300. 15366300

[16] Hosseini K. , Akhondian S. , Jafarpour K. , Tolooei A. , Valizadeh M. , and Ganjehzadeh S. , Management and Treatment Modalities of Inferior Alveolar Nerve Injuries: Review of Literature, Oral Science International. (2025) 22, no. 1, e1272, 10.1002/osi2.1272.

[17] Byun S. H. , Kim S. S. , Chung H. J. , Lim H. K. , Hei W. H. , Woo J. M. , Kim S. M. , and Lee J. H. , Surgical Management of Damaged Inferior Alveolar Nerve Caused by Endodontic Overfilling of Calcium Hydroxide Paste, International Endodontic Journal. (2016) 49, no. 11, 1020–1029, 10.1111/iej.12560, 2-s2.0-85027932147, 26537746. 26537746

[18] Schwenzer N. and Ehrenfeld M. , Zahn-Mund-Kiefer-Heilkunde. Mund-Kiefer-Gesichtschirurgie, 2011, Thieme, 10.1055/b-002-15441.

[19] Denio D. , Torabinejad M. , and Bakland L. K. , Anatomical Relationship of the Mandibular Canal to Its Surrounding Structures in Mature Mandibles, Journal of Endodontics. (1992) 18, no. 4, 161–165, 10.1016/S0099-2399(06)81411-1, 2-s2.0-0026847059, 1402570. 1402570

