# Supplementary material for: Invasion of Calcium Hydroxide Preparations Leading to Severe Chemical Nerve Injury Treated Through Nerve Repair Using Artificial Nerve Conduit: A Case Report
Source: Case Rep Dent. 2025 Dec 12;2025:6610331. doi: 10.1155/crid/6610331 (PMC12721732; doi:10.1155/crid/6610331)
Supplement: Supplementary file 1 — Supporting Information Additional supporting information can be found online in the Supporting Information section. We used the CARE‐checklist‐English‐2013 as a reporting guideline. [file CRID-2025-6610331-s001.pdf]

CARE
case report guidelines

CARE Checklist of information to include when writing a case report

(cc) BY-NC-ND

Topic	Item	Checklist item description	Reported on Line
Title	1	The diagnosis or intervention of primary focus followed by the words "case report"	lines 1 to 2
Key Words	2	2 to 5 key words that identify diagnoses or interventions in this case report, including "case report"	line 41
Abstract (no references)	3a	Introduction: What is unique about this case and what does it add to the scientific literature?	lines 21 to 25
	3b	Main symptoms and/or important clinical findings	lines 26 to 27
	3c	The main diagnoses, therapeutic interventions, and outcomes	lines 28 to 33
	3d	Conclusion—What is the main "take-away" lesson(s) from this case?	lines 34 to 38
Introduction	4	One or two paragraphs summarizing why this case is unique (**may include references**)	lines 44 to 66 (2 paragraph)
Patient Information	5a	De-identified patient specific information.	lines 69 to 70
	5b	Primary concerns and symptoms of the patient.	lines 69 to 73
	5c	Medical, family, and psycho-social history including relevant genetic information	line 73
	5d	Relevant past interventions with outcomes	lines 71 to 76
Clinical Findings	6	Describe significant physical examination (PE) and important clinical findings.	lines 75 to82
Timeline	7	Historical and current information from this episode of care organized as a timeline	lines 69 to 95, and Table 1
Diagnostic Assessment	8a	Diagnostic testing (such as PE, laboratory testing, imaging, surveys).	lines75 to 80
	8b	Diagnostic challenges (such as access to testing, financial, or cultural)	N/A
	8c	Diagnosis (including other diagnoses considered)	lines 73, 74
	8d	Prognosis (such as staging in oncology) where applicable	lines 87 to 89
Therapeutic Intervention	9a	Types of therapeutic intervention (such as pharmacologic, surgical, preventive, self-care)	lines 116 to 119
	9b	Administration of therapeutic intervention (such as dosage, strength, duration)	lines 116 to 119
	9c	Changes in therapeutic intervention (with rationale)	lines 85 to 95
Follow-up and Outcomes	10a	Clinician and patient-assessed outcomes (if available)	lines 120 to 124
	10b	Important follow-up diagnostic and other test results	lines 120 to124
	10c	Intervention adherence and tolerability (How was this assessed?)	lines 120 to 124
	10d	Adverse and unanticipated events	lines 119, none
Discussion	11a	A scientific discussion of the strengths AND limitations associated with this case report	lines 126 to 189
	11b	Discussion of the relevant medical literature **with references**.	including reference no.2-18
	11c	The scientific rationale for any conclusions (including assessment of possible causes)	line 127 to 189
	11d	The primary "take-away" lessons of this case report (without references) in a one paragraph conclusion	linse 183 to 189
Patient Perspective	12	The patient should share their perspective in one to two paragraphs on the treatment(s) they received	lines 151 to 183
Informed Consent	13	Did the patient give informed consent? Please provide if requested	Yes ☑ No ☐
